# Effects of calcitriol on random skin flap survival in rats

**DOI:** 10.1038/srep18945

**Published:** 2016-01-06

**Authors:** Kai-liang Zhou, Yi-hui Zhang, Ding-sheng Lin, Xian-yao Tao, Hua-zi Xu

**Affiliations:** 1 Department of Orthopaedic Surgery, The Second Affiliated Hospital of Wenzhou Medical University & The Second Clinical Medical College of Wenzhou Medical University, Wenzhou, China; 2 Department of Traditional Chinese Medicine, The Second Affiliated Hospital of Wenzhou Medical University & The Second Clinical Medical College of Wenzhou Medical University, Wenzhou, China

## Abstract

Calcitriol, a metabolite of vitamin D, is often used in osteoporosis clinics. However, the material has other bioactivities; for example, it accelerates angiogenesis, has anti-inflammatory properties, and inhibits oxidative stress. We investigated the effects of calcitriol in a random skin flap rat model. “McFarlane flap” models were established in 84 male Sprague Dawley rats, divided into two groups. One group received intraperitoneal injections of calcitriol (2 μg/kg/day) whereas control rats received intraperitoneal injections of saline. The percentage flap survival area and tissue water content were measured 7 days later, which showed that calcitriol improved flap survival area and reduced tissue edema. It also increased the mean vessel density and upregulated levels of VEGF mRNA/protein, both of which promote flap angiogenesis. Moreover, it decreased leukocyte and macrophage infiltration, reduced the inflammatory proteins IL1β and IL6, increased SOD activity, decreased MDA content, and upregulated the level of autophagy. Overall, our results suggest that calcitriol promotes skin flap survival by accelerating angiogenesis, having anti-inflammatory effects, reducing oxidative stress, and promoting autophagy.

Random skin flap transplantation is used frequently in plastic surgery^1^. However, distal flap necrosis remains challenging. Although flap design and surgical techniques have improved over the years, the length-to-width ratio cannot be >1.5-2:1, limiting the clinical applications of such flaps. Previous studies found that inadequate blood supply^2^, inflammatory reactions^3^, and oxidative stress^4^ are three important factors contributing to flap necrosis.

Calcitriol (C_27_H_44_O_3_), also known as “1,25-dihydroxyvitamin D_3_”, exerts many functions associated with bone calcium metabolism and plays a key role in osteoporosis. However, calcitriol has recently been shown to exhibit various other bioactivities. For example, it increases vascular endothelial growth factor (VEGF) expression by binding to a vitamin D response element in the VEGF promotor^5^. Moreover, VEGF levels in vascular endothelial cells are specifically affected; the cells are stimulated to proliferate and regenerate, promoting angiopoiesis^6^. Calcitriol has also recently been shown to exert anti-inflammatory effects in a model of diabetic nephropathy^7^. Sezgin *et al.*^8^ showed that 1,25-dihydroxyvitamin D_3_ reduced oxidative stress status in a model of renal ischemia-reperfusion injury. It has been demonstrated that calcitriol stimulates autophagy^9^, which relieves oxidative stress^10^. Moreover, autophagy is a process whereby cells degrade cytosolic macromolecules and organelles in lysosomes, and is thus generally considered to be a survival tactic protecting against stress (e.g., starvation, pro-oxidant conditions)^11^.

Hence, we hypothesized that calcitriol might enhance the survival of random skin flaps. The anti-inflammatory properties of calcitriol, together with its ability to accelerate vascularization, suppress oxidative stress, and induce autophagy, should be helpful in this context. We investigated whether calcitriol exerted such effects in a random skin flap model, via histological and protein analyses.

## Results

### Calcitriol improves flap survival area and reduces tissue edema

On the first day after the operation, the flaps of both groups were pale and swollen to some extent. Both Areas III exhibited edema and were grey/purple in color, without obvious necrosis. On day 3, Area II and III of all flaps were darker and some areas of necrosis had appeared, associated with a brown nidus. On day 7, Area I of all flaps had survived, whereas Area III had become darker with necrosis spreading to Area II, with scabbing and hardening, in both control and calcitriol groups. Boundaries were evident between the surviving and necrotic regions.

**Figure 1:**
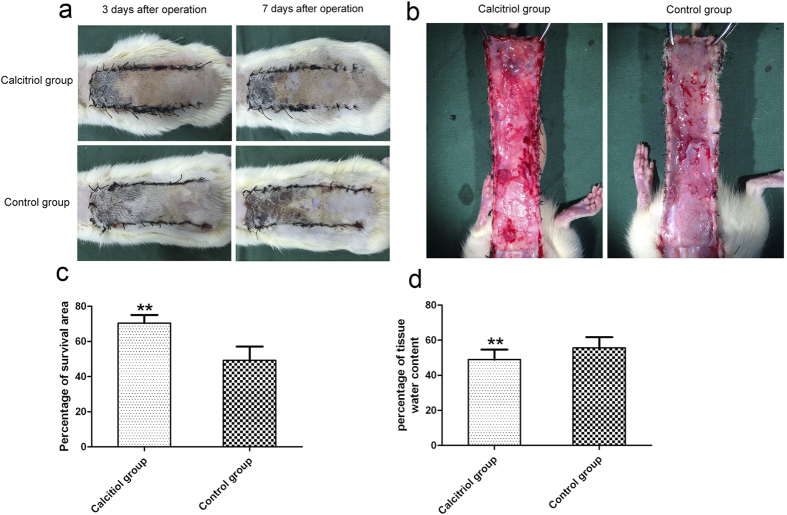
Calcitriol improves flap survival area and reduces tissue edema. (**a**) Digital photographs show the postoperative flaps of the calcitriol and control groups on Days 3 and 7. (**b**) Digital photographs show the tissue edema of postoperative flaps of each group on Day 7. (**c**) Histogram of percentages of survival area in the calcitriol group (70.42 ± 4.16%) and control group (49.20 ± 4.30%). (**d**) Histogram of percentages of tissue water content: 46.90 ± 5.45% in the calcitriol group and 57.45 ± 3.05% in the control group. Values are expressed as the mean ± SEM, *n* = 6 per group. **p <* *0.01, vs. control group.

**Figure 2:**
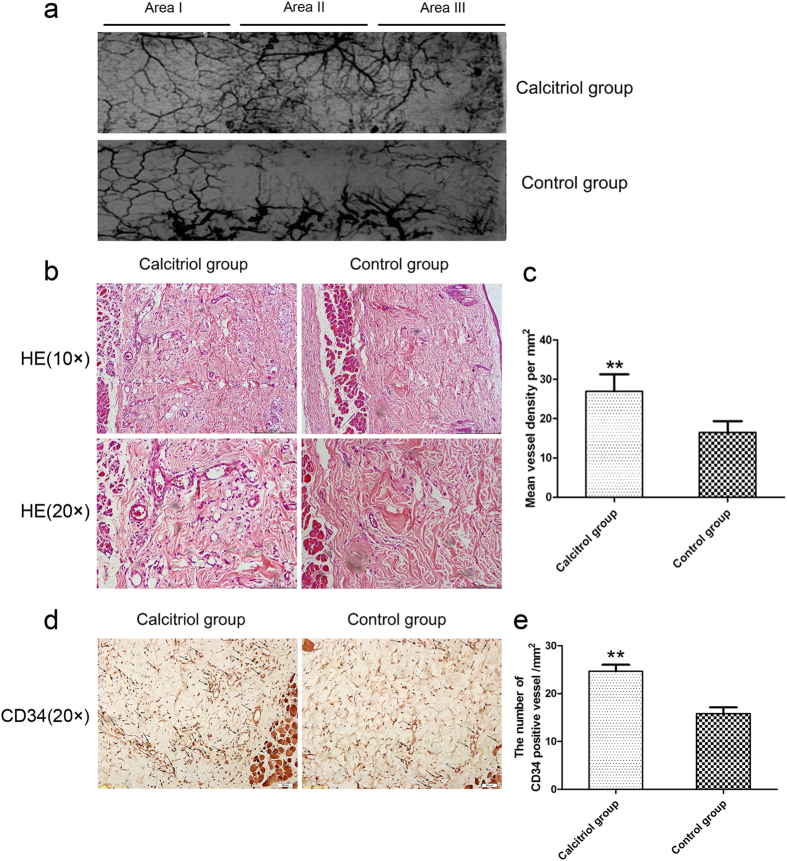
Calcitriol promotes vascularization in skin flaps. (**a**) Flap angiograms on postoperative Day 7 after surgery. (**b**) Neovascularization in calcitriol and control groups by H&E staining (original magnification ×100 and ×200). (**c**) Histogram of percentages of MVDs: calcitriol group (26.96 ± 4.33/mm^2^) and control group (16.48 ± 2.87/mm^2^). (**d**) CD34-positive vessels in the calcitriol and control groups as assessed by immunohistochemistry (original magnification ×200). (**e**) The numbers of CD34-positive vessels/mm^2^ were 24.67 ± 3.89/mm^2^ in the calcitriol group and 15.83 ± 3.19/mm^2^ in the control group. Values are expressed as means ± SEM, *n* = 6 per group. **p <* *0.01, vs. control group.

Survival of Area II in the calcitriol group was better than that of the control group, with less necrosis (Fig. 1a). The mean surviving areas were 70.42 ± 4.16% and 49.20 ± 4.30% in the calcitriol and control groups, respectively. The calcitriol (treated) fraction was significantly higher than the control (Fig. 2c; *p* < 0.01). Percent tissue water content was significantly lower in the calcitriol group (46.90 ± 5.45%) than in the control group (57.45 ± 3.05%; Fig. 1b,d; *p *<* *0.01), indicating that tissue edema was lower in the former.

### Calcitriol promotes vascularization in skin flaps

**Figure 3:**
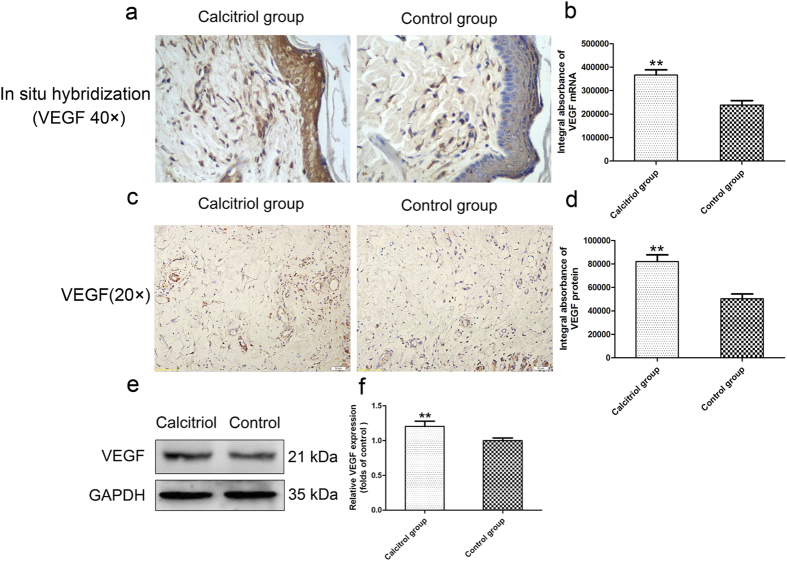
Calcitriol increases levels of VEGF mRNA/protein in skin flaps. (**a**) *In situ* hybridization for VEGF mRNA in the calcitriol and control groups (original magnification × 400). (**b**) The integral absorbance (IA) values for VEGF mRNA were 366624.00 ± 50300.32 in the calcitriol group and 238306.20 ± 43730.12 in the control group. (**c**) VEGF expression in each group as assessed by immunohistochemistry (original magnification × 200). (**d**) The IA values of VEGF protein were 82087.16 ± 12687.08 in the calcitriol group and 50490.62 ± 8883.89 in the control group. (**e**) Protein expression of VEGF in each group, as assessed by Western blot analysis. The gels have been run under the same experimental conditions, and cropped blots are used here. The full-length gel images are available in Supplementary Fig. 3e. (**f**) Densitometry results of VEGF protein expression in the two groups. Values are expressed as means ± SEMs, *n* = 6 per group. **p < 0.01, vs. control group.

On day 7 after surgery, all flaps in the calcitriol and control groups were morphologically similar. Although Area I survived, and necrosis was evident to the naked eye in Area III, Area II of the test and control flaps differed. As is shown in X-ray images, microvessels of flaps in the backs of rats in the two groups were well perfused and clear. There was almost no vascular imaging in Area I of flaps in either group. However, the microvascular imaging range of Area II and Area III was significantly greater in the calcitriol group than in the control group (Fig. 2a). Calcitriol group flaps exhibited more neovascularization, more subcutaneous hemorrhaging, and less necrosis than control flaps (hematoxylin and eosin staining; Fig. 2b). The mean vessel densities (MVDs) of Area II in the two groups from the results of H&E staining were 26.96 ± 4.33/mm^2^ and 16.48 ± 2.87/mm^2^, respectively (Fig. 3c; *p* < 0.01). CD34 is usually used to label endothelial cells. Thus, the MVDs of Area II in the two groups were also reflected directly by the number of CD34-positive vessels/mm^2^. As shown in Fig. 2d, calcitriol-treatment increased the number of CD34-positive vessels in the random skin flap model: there were 24.67 ± 3.89/mm^2^ in the calcitriol group and 15.83 ± 3.19/mm^2^ in the control group (Fig. 2e; *p* < 0.01).

### Calcitriol increases levels of VEGF mRNA/protein in skin flaps

*In situ* hybridization for VEGF mRNA in Area II of the two groups was performed. As shown in Fig. 3a, more VEGF mRNA was synthesized by keratinocytes and fibroblasts in cutis and dermal vascular structures in the calcitriol group than in the control group. Moreover, based on calculations of the IA, the levels of VEGF mRNA in the calcitriol and control groups were 2002.26 ± 203.76 and 970.18 ± 171.75, respectively (Fig. 3b; *p* < 0.01). Immunohistochemical staining for VEGF protein was performed to distinguish the cells expressing this protein. As shown in Fig. 3c, VEGF was expressed in vessels and stromal cells in the dermis of random skin flaps of the two groups; clearly more VEGF expression was observed in the calcitriol group. The IA values of VEGF protein in the calcitriol group and control group were 82087.16 ± 12687.08 and 50490.62 ± 8883.89, respectively (Fig. 3d; *p* < 0.01). Western blot analysis also showed that the calcitriol group expressed more VEGF than the control group (Fig. 3e,f; *p* < 0.01).

### Calcitriol suppresses inflammation in skin flaps

**Figure 4:**
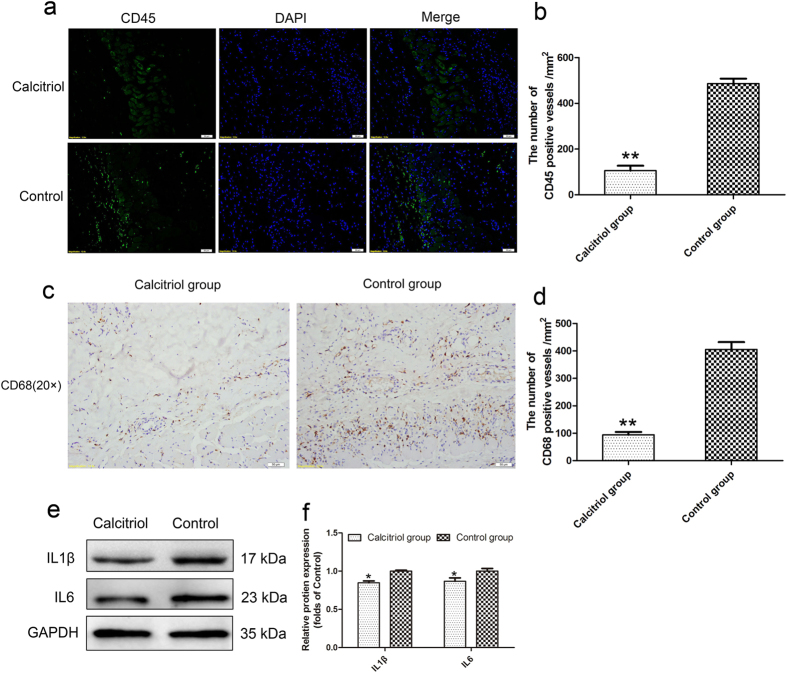
Calcitriol suppresses inflammatory response in skin flaps. (**a**) Leukocyte infiltration in the calcitriol and control groups as assessed by immunofluorescence for CD45 (original magnification × 200). (**b**) The numbers of CD45-positive cells/mm^2^ were 105.71 ± 48.02/mm^2^ in the calcitriol group and 486.67 ± 48.52/mm^2^ in the control group. (**c**) Macrophage infiltration in each group as assessed by immunohistochemistry staining for CD68 (original magnification ×200). (**d**) The numbers of CD68-positive cells/mm^2^ were 94.49 ± 22.86/mm^2^ in the calcitriol group and 405.71 ± 59.35/mm^2^ in the control group. (**e**) Protein expression levels of IL1β and IL6 in each group as assessed by Western blot analysis. The gels have been run under the same experimental conditions, and cropped blots are used here. The full-length gel images are available in Supplementary Fig. 4e. (**f**) Optical densities of IL1β and IL6 proteins. Values are expressed as means ± SEMs, *n* = 6 per group. **p < 0.01, vs. control group, *p < 0.05, vs. control group.

Immunofluorescence (IF) staining for CD45 (a common leukocyte marker) and immunohistochemical staining for CD68 (a macrophage marker) were performed to determine the inflammatory response in the random skin flaps. Under the fluorescence microscope, Area II of test flaps exhibited less leukocyte infiltration than Area II of control flaps (Fig. 4a). The mean numbers of leukocytes/mm^2^ were 105.71 ± 48.02/mm^2^ in the calcitriol group and 486.67 ± 48.52/mm^2^ in the control group (Fig. 4b; *p* < 0.01). As shown in Fig. 4c, macrophage infiltration was less in the calcitriol group than in the control group, as assessed by immunohistochemistry for CD68. The numbers of CD68-positive cells/mm^2^ were 94.49 ± 22.86/mm^2^ in the calcitriol group and 405.71 ± 59.35/mm^2^ in the control group (Fig. 4d; *p* < 0.01). Expression levels of IL1β and IL6 were lower in the calcitriol group than in the control group (Fig. 4e,f; *p* < 0.05).

### Calcitriol attenuates oxidative stress in skin flaps

**Figure 5:**
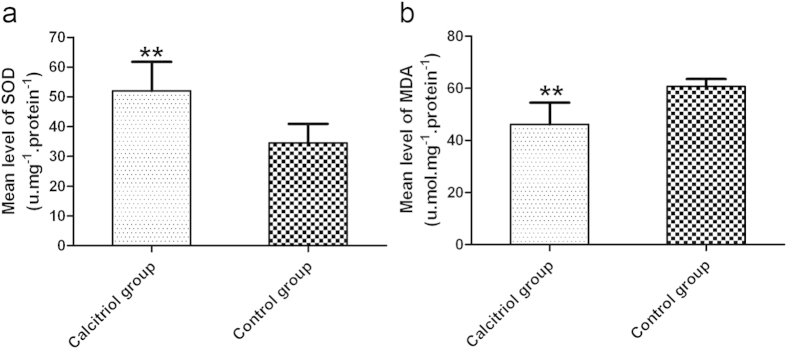
Calcitriol attenuates oxidative stress in skin flaps. (**a**) The levels of superoxide dismutase activity were 52.00 ± 9.76 U.mg^−1^protein^−1^ in the calcitriol group and 34.50 ± 6.44 U.mg^−1^protein^−1^ in the control. (**b**) Treatment with calcitriol resulted in a malondialdehyde content of 46.12 ± 8.33 nmol.mg^−1^protein^−1^ in the calcitriol group and 60.67 ± 2.88 nmol.mg^−1^protein^−1^ in the control group. Values are expressed as means ± SEMs, *n* = 6 per group. **p < 0.01, vs. control group.

The calcitriol group had a much higher mean level of superoxide dismutase (SOD) (52.00 ± 9.76 U.mg^−1^ protein^−1^) than the control group (34.50 ± 6.44 U.mg^−1^ protein^−1^) (Fig. 5a; *p *<* *0.01). The mean level of malondialdehyde (MDA) in the test group was 46.12 ± 8.33 nmol.mg^−1^ protein^−1^, significantly less than 60.67 ± 2.88 nmol.mg^−1^ protein^−1^ in the control group (Fig. 5b; *p* < 0.01).

### Calcitriol upregulates autophagy in skin flaps

**Figure 6:**
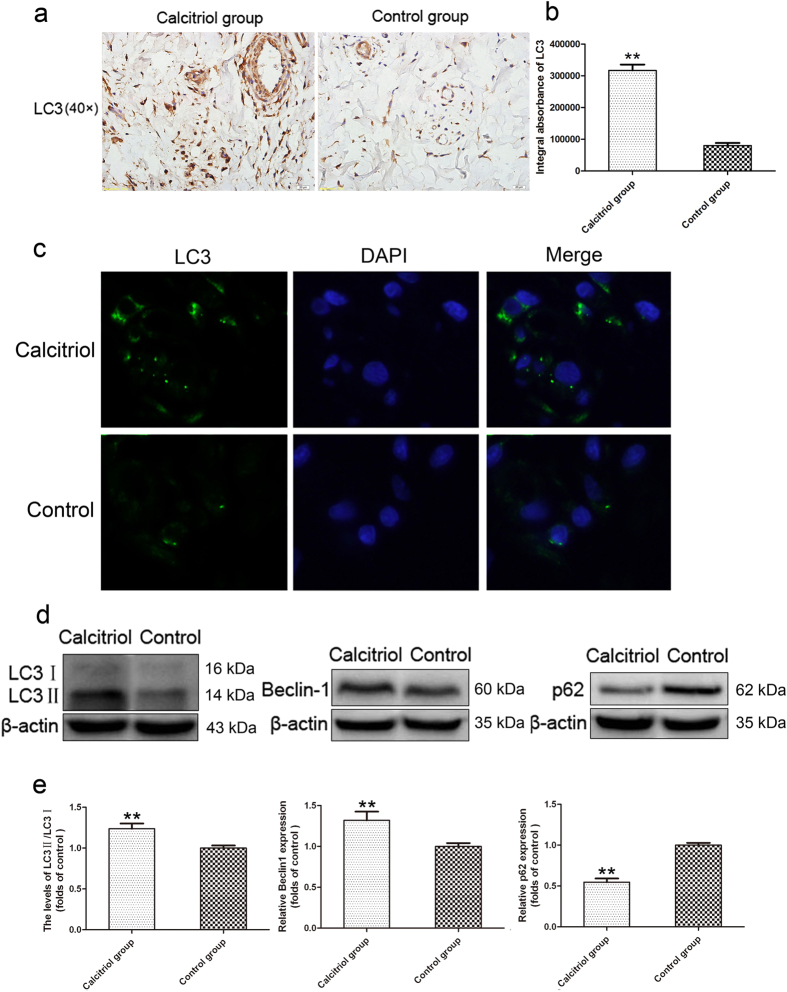
Calcitriol upregulates autophagy in skin flaps. (**a**) LC3II punctate dots were seen in the calcitriol and control groups in immunohistochemistry assessments (original magnification ×400). (**b**) Integral absorbance (IA) values for LC3 were 317103.53 ± 45034.03 in the calcitriol group and 80303.13 ± 19882.24 in the control group. (**c**) Immunofluorescence for LC3II punctate dots in the calcitriol group and control group. (**d**) Protein expression of LC3, Beclin1, and p62 in each group as assessed by Western blot analysis. The gels have been run under the same experimental conditions, and cropped blots are used here. The full-length gel images are available in Supplementary Fig. 6d. (**e**) Optical density analysis of ratios of LC3II/LC3I, Beclin1, and p62 protein expression in the calcitriol and control groups. Values are expressed as means ± SEMs, *n* = 6 per group. **p < 0.01, vs. control group.

Compared to flap cells in the control group, there were more LC3II punctate dots in the cytoplasm of flap cells with calcitriol treatment (Fig. 6a). Based on calculations of the IA, the levels of LC3 expression were 317103.53 ± 45034.03 and 80303.13 ± 19882.24 in the calcitriol and control groups, respectively (Fig. 6b; *p* < 0.01). IF staining was also performed to label LC3II/DAPI (Fig. 6c). The results further confirmed that calcitriol treatment increased the LC3II-positive dots in the cytoplasm of flap cells. Western blotting detected LC3II/LC3I and Beclin1 expression in Area II of all flaps (Fig. 6d). The LC3II/LC3I ratio and Beclin1 expression were significantly greater in the calcitriol group than in the control group (Fig. 6e; *p* < 0.01). These results indicate that more autophagosomes were generated in the cytoplasm of flap cells with calcitriol treatment. However, the generation of autophagosomes does not indicate activation of the autophagic process. Autophagy is a dynamic mechanism of degradation of damaged cellular organelles and long-lived proteins. Protein p62 is a substrate of the autophagic process, and its level is a marker of autophagic flux. The expression level of p62 in the calcitriol group was detected by Western blotting, and was much lower than in the control group (Fig. 6d,e; *p *<* *0.01).

## Discussion

Calcitriol, also known as 1,25-dihydroxyvitamin D_3_, is the biologically active metabolite of vitamin D and the principal Ca^2+^-regulatory steroid hormone^12^. Calcitriol plays several roles in osteoporosis. However, many studies have also shown that calcitriol has other bioactivities, such as anti-inflammatory^13^ and anti-neoplastic^14^ properties, and promotion of vascularisation^15^ and so on. Thus, we hypothesized that calcitriol would enhance random skin flap viability by promoting vascularization, suppressing inflammation, and attenuating oxidative stress.

Previous studies have shown that calcitriol treatment increases the expression of VEGF in breast cancer and skeletal muscle cells^16^. VEGF specifically affects vascular endothelial cells, stimulating proliferation and regeneration and thus promoting angiopoiesis^6^. In a lung cancer model, angiopoiesis in lung carcinoma cells was reduced by anti-VEGF therapy; the cancer was ‘cured’^17^. In skin flaps, VEGF is secreted by keratinocytes and fibroblasts in the cutis, and it is especially active in dermal vascular structures^18^. Vascularization of random skin flaps is promoted by administration of VEGF^19^. In addition, hypoxic keratinocytes synthesize mRNAs encoding VEGF-121 and VEGF-156, soluble isoforms that diffuse through several cell layers and the basal lamina to their targets (receptors on the surface of the dermal vascular endothelium)^20^. In our research, VEGF mRNA synthesis in keratinocytes in the cutis of skin flaps was increased after calcitriol treatment. Moreover, levels of VEGF mRNA/protein in vessels and stromal cells in the dermis were both upregulated after calcitriol treatment. Western blotting also revealed higher levels of VEGF in the calcitriol group than the control group. Furthermore, the MVD results from H&E staining and CD34 staining both showed more neovascularization in the calcitriol group than in the control group. Thus, we conclude that calcitriol induces vascularization in ischemic skin flaps by upregulating VEGF protein/mRNA levels.

Inflammation plays an important role in the survival of random skin flaps; moderate coagulative necrosis with inflammatory cell infiltration is evident in the epidermis of random skin flaps. The greater the extent of necrosis, the more pronounced the inflammation, which compromises flap success^21^. When inflammatory responses are exacerbated, attenuation of the inflammation ameliorates healing. Calcitriol has recently been shown to have anti-inflammatory effects in a bullous pemphigoid model^22^. Consistent with other studies, our results demonstrate that calcitriol reduces the inflammatory response, as reflected by decreased IL-6 levels^23^ and monocyte/macrophage activation^24^. We also found that IL1β levels and leukocyte invasion were reduced in random skin flaps treated with calcitriol. Thus, we conclude that calcitriol has strong anti-inflammatory activities in the random skin flap model.

Ischemia-reperfusion injury involves a complex oxidation process and is closely related to random skin flap survival^25^. Its many significant components include the generation of reactive oxygen species (ROS). In the early stages of oxidative stress, these radicals react with the lipids of cell membranes and proteins, triggering peroxidation and destroying cells and tissues. MDA is a marker of lipid peroxidation, and its levels reflect the extent of tissue injury^26^. SOD is one of the body’s defenses against oxygen free radicals. The SOD level is an indicator of antioxidant status; the enzyme clears O^2−^ radicals and prevents tissue injury. Thus, SOD activity and MDA content are important biomarkers of oxidative stress status. Calcitriol protects against ischemia-reperfusion injury in the rat hippocampus^27^. In this study, SOD activity was much higher in the calcitriol group than in the control group, and the MDA level was lower. Thus, calcitriol suppresses oxidative stress in random skin flaps.

Generally, ROS are believed to induce angiogenesis via several known pathways, including the Nox1/SHP-1^28^ and CEP/TLR2 pathways^29^. However, a recent paper indicated that an excessive amount of ROS, induced by ATM deficiency, inhibited angiogenesis^30^. Thus, the effects of ROS on angiogenesis remain to be determined. The effect is likely to depend on the conditions characteristic of a given disease. In a model of random skin flaps, Suzuki *et al.*^31^ suggested that the generation of ROS contributed to flap necrosis, and treatment with liposomal SOD decreased distal flap necrosis. In the present study, we found that calcitriol enhanced the survival of random skin flaps by accelerating vascularization and suppressing oxidative stress. However, whether suppressing oxidative stress accelerates or suppresses vascularization in random skin flaps after calcitriol-treatment remains to be further researched.

In recent years, it has been reported that calcitriol upregulates autophagy and even induces autophagy in SH-SY5Y cells (a model of Parkinson’s disease)^9^. Autophagy is the process whereby cells degrade cytosolic macromolecules and organelles in lysosomes. Autophagy is generally considered to be a survival tactic using to protect against stress (e.g., starvation, pro-oxidant conditions)^11^. Autophagy has been shown to have a protective effect in many animal and tissue models (including models of AD^32^ and spinal cord injury^33^). However, any role played by autophagy in the skin flap model was unclear. To our knowledge, this is the first report of calcitriol-mediated activation of autophagy in random skin flaps. In this study, both immunohistochemistry and IF revealed that more LC3II punctate dots were generated in the cytoplasm of flap cells with calcitriol treatment. Furthermore, Western blotting showed that LC3II/LC3I and Beclin1 increased, indicating that autophagy vesicles were enhanced in the calcitriol group. However, the generation of autophagosomes does not indicate activation of the autophagic process, which is a flux. Autophagy is a dynamic mechanism of degradation of damaged cellular organelles and long-lived proteins. The protein p62 is a substrate of the autophagic process; thus, its level is a marker of autophagic flux. In our research, the level of p62 protein was detected by Western blotting. Compared to the control group, p62 was significantly decreased, indicating that autophagy flux was enhanced in the calcitriol group. Thus, calcitriol appears to upregulate the level of autophagy in random skin flaps.

In the present study, calcitriol enhanced random skin flap survival apparently by suppressing oxidative stress. Calcitriol may also increase the level of autophagy. Increasing evidence shows that oxidative stress is reduced when autophagy is upregulated. ROS are generated by damaged mitochondria under conditions of oxygen stress, and excess ROS oxidatively damage other cellular components. Autophagy sequesters and degrades damaged mitochondria, helping cells to escape death^34^. When autophagy is inhibited, damaged mitochondria accumulate and produce more ROS^35^, ultimately triggering necrosis. Tian *et al.*^36^ found that autophagy was required to maintain healthy mitochondria and to reduce oxidative stress, preventing the initiation of hepatocarcinogenesis. Thus, autophagy can reduce both oxidative stress and associated injuries. Based on our results, we suggest that calcitriol may reduce oxidative stress by upregulating autophagy in random skin flaps.

In conclusion, calcitriol increased angiogenesis, suppressed inflammatory reactions, and reduced oxidative stress, contributing to a significant increase in random skin flap survival. Furthermore, autophagy increased in skin flaps treated with calcitriol; this may reduce oxidative stress. Further experimental and clinical studies on calcitriol are needed.

## Materials and Methods

### Animals

Healthy male Sprague Dawley rats (250–300 g) were purchased from Wenzhou Medical University (license no. SCXK[ZJ]2005-0019). All animal care and use conformed to the Guide for the Care and Use of Laboratory Animals of the Chinese National Institutes of Health and the work was approved by the Animal Care and Use Committee of Wenzhou Medical University (wydw2012-0079). The rats were divided randomly into two groups: a calcitriol group (experimental group) and a saline group (control group). Each group contained 42 rats.

### Flap animal model

Rats were anesthetized by administration of 2% (w/v) pentobarbital sodium (40 mg/kg, Solarbio Science & Technology, Beijing, China) via intraperitoneal injection. A modified McFarlane flap model was created in the rat dorsum (in the same position in all rats)^37^. We outlined caudal 3 × 9 cm skin/panniculus carnosus flaps on the back of each rat and sectioned both sacral arteries. Each flap was completely separated from the underlying fascia and immediately sutured to the donor bed using 4-0 silk and a wedged-on cutting needle. The flap area was divided into three equal zones: proximal (Area I), intermediate (Area II), and distal (Area III).

### Experimental protocol

Calcitriol (Cayman, Ann Arbor, MI, USA) was dissolved in ethanol (1 mg/mL) and further diluted in saline immediately prior to intraperitoneal (i.p.) administration. The calcitriol group (*n* = 42) received calcitriol at 2 μg/kg/day on 7 consecutive days. The saline group (*n* = 42) received equal volumes of saline supplemented with the same amount of ethanol for 7 days. The first drug injection was given 2 h after the surgical procedure. All animals were housed individually in standard experimental cages in an environmentally controlled room and were provided with standard rat chow and water *ad libitum*. Each rat was fitted with a neck collar (Fig. S1) to prevent self-mutilation. All rats were sacrificed with an overdose of pentobarbital sodium at 7 days.

### General observation and flap assessment

Flap survival was observed, and macroscopic changes developing during the 7 days, including appearance, color, texture, and hair condition, were noted. On postoperative day 7, the surviving flap areas were measured by superimposition of photographs on graph paper. All results are expressed as percentages of viable area calculated as: extent of viable area × 100%/total area (viable and ischemic).

### Hematoxylin and eosin (H&E) staining

Three samples (1 cm × 1 cm) of central tissue from each flap Area (see above) were collected and biopsied after sacrifice. Samples (1 cm × 1 cm) were post-fixed in 4% (v/v) paraformaldehyde for 24 h and embedded in paraffin wax for transverse sectioning. The sections (4 μm thick) were mounted on poly-L-lysine-coated slides for hematoxylin and eosin staining. We measured the thickness of granulation tissue, tissue edema, and leukocyte infiltration under a light microscope (×100 and ×200 magnification), and calculated the number of microvessels per unit area (/mm^2^) (an indicator of microvascular density).

### Tissue edema measurement

Tissues edema was reflected by water content. At 7 days after operation, flap tissues were weighed and then dehydrated in an autoclave at 50 °C. We weighed all samples daily until the weight did not change for 2 days. The percentage water content was determined as follows:


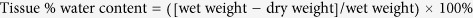
.

### Flap angiography

Seven days after the operation, six rats in each group underwent whole-body angiography according to a modified lead oxide-gelatin (Shanghai Chemical, Shanghai, China) injection technique with a 24-gauge intravenous silicone catheter. The right common carotid artery was injected with 1.5 mL 1% heparin saline, followed by injection of 150 mL/kg contrast medium (a mixture of lead oxide, gelatin, and water). After 24 h of fixation, the flaps were obtained and radiographed (54 kVp, 40 mA, 100 s exposure) with a soft X-ray machine.

### Superoxide dismutase activity and malondialdehyde content

Superoxide dismutase (SOD) and malondialdehyde (MDA) test kits (Nanjing Jiancheng Biology Institution, Nanjing, China) were used to measure oxidative stress status of the flaps. On day 7 postoperatively, 10 tissue specimens (0.5 cm × 0.5 cm) were obtained from Area II of each group, weighed, homogenized, and diluted to 10% (v/v) in an ice bath. Superoxide dismutase (SOD) activity was determined using the xanthine oxidase method, and malondialdehyde (MDA) content was measured via reaction with thiobarbituric acid (TBA) at 90–100 °C^38^.

### Immunohistochemistry

Six section specimens of Area II in each group were deparaffinized in xylene and rehydrated through a graded set of ethanol baths. After washing, the sections were blocked with 3% (v/v) H_2_O_2_ and treated with 10.2 mM sodium citrate buffer (antigen retrieval) for 20 min at 95 °C. After blocking with 5% (w/v) bovine serum albumin and 1% (v/v) Tween-20 in PBS for 10 min, the sections were incubated with antibody against CD34 (1:100, Abcam, Cambridge, MA, USA), CD68 (1:150, Abcam), VEGF (1:500; Bioworld, Nanjing, China), LC3 (1:400; Cell Signaling Technology; Danvers, MA, USA) overnight at 4 °C. Finally, the sections were incubated with an appropriate HRP-conjugated secondary antibody (Santa Cruz Biotechnology, Dallas, TX) and counterstained with hematoxylin. Flap tissues were imaged at ×200/×400 magnification using a DP2-TWAN image-acquisition system (Olympus Corp). Observation parameters (white balance, aperture, shutter speed, and time) were held constant. Images were saved using the Image-Pro Plus software (ver. 6.0; Media Cybernetics, Rockville, MD) and integral absorbance (IA) values were used as indicators of VEGF and LC3 expression levels. The numbers of CD34-positive blood vessels and CD68-positive cells per unit area (mm^2^) were calculated. Six random fields of three random sections from each tissue sample were used to quantify the positive cells.

### Immunofluorescence

Six section specimens of Area II in each group were deparaffinized in xylene and rehydrated through a graded set of ethanols. After washing, the sections were treated with 10.2 mM sodium citrate buffer (antigen retrieval) for 20 min at 95 °C. Then the sections were permeabilized with 0.1% (v/v) PBS-Triton X-100 for 30 min. After blocking in 10% (v/v) bovine serum albumin in PBS for 1 h, slides were incubated at 4 °C overnight with a primary antibody against CD45 (1;200; Abcam) or LC3 (1:200; Cell Signal Technology). Then the slides were washed three times for 10 min at room temperature, and incubated with fluorescein isothiocyanate (FITC)-conjugated goat anti-rabbit IgG (1:200) antibody for 1 h at room temperature. All images were evaluated under a fluorescence microscope (Olympus, Tokyo, Japan). The number of CD45-positive cells per unit area (mm^2^) was calculated. Six random fields of three random sections from each tissue sample were used.

### *In situ* hybridization

A VEGF mRNA *in situ* hybridization kit (Boster Inc., Wuhan, China) was used to detect the level of VEGF mRNA. The probe sequences were 5′-GCTCT ACCTC CACCA TGCCA AGTGG TCCCA-3′, 5′-GACCC TGGTG GACAT CTTCC AGGAG TACCC-3′, and 5′-GCAGC TTGAG TTAAA CGAAC GTACT TGCAG-3′. The procedure was carried out according to the kit instructions. After staining with DAB, the sections were dehydrated with graded ethanols, mounted with xylene, and sealed. Then the flap tissues were imaged at ×400 magnification using a DP2-TWAN image-acquisition system (Olympus Corp). Observation parameters (white balance, aperture, shutter speed, and time) were held constant. Images were saved using the Image-Pro Plus software (ver. 6.0; Media Cybernetics) and the IA values were used as indicators of VEGF mRNA levels. Six random fields of three random sections from each tissue sample were used.

### Western blotting

On day 7 after surgery, tissues (1 cm × 1 cm) from Area II were dissected and stored at −80 °C prior to Western blotting. Protein concentrations were determined using the BCA assay (Thermo, Rockford, IL, USA). Seventy microgram amounts of protein were separated on a 12% (w/v) gel and transferred onto PVDF membranes (Roche Applied Science, Indianapolis, IN). After blocking with 5% (w/v) non-fat milk for 2 h, the membranes were incubated with antibodies against VEGF (1:400; Bioworld, Nanjing, China), IL1β, IL6, GAPDH (1:1000; Abcam), Beclin1, p62, LC3 (1:1000; Cell Signaling Technology), and β-actin (1:200; Santa Cruz Biotechnology). Next, the membranes were incubated with a goat-anti-rabbit secondary antibody for 2 h at room temperature and bands detected using the ECL-plus reagent kit (PerkinElmer, Waltham, MA, USA). Band intensity was quantified using the Image Lab 3.0 software (Bio-Rad).

### Statistical analysis

Statistical analyses were performed using the SPSS software (ver. 19.0; SPSS, Chicago, IL). Data are expressed as means ± SEMs. Statistical evaluations were done using Student’s *t*-test. In all analyses, *p* values < 0.05 were considered to indicate statistical significance.

## Additional Information

**How to cite this article**: Zhou, K.-l. *et al.* Effects of calcitriol on random skin flap survival in rats. *Sci. Rep.* **6**, 18945; doi: 10.1038/srep18945 (2016).

## Supplementary Material

Supplementary Information
