# Supplementary material for: Effects of calcitriol on random skin flap survival in rats
Source: Sci Rep. 2016 Jan 6;6:18945. doi: 10.1038/srep18945 (PMC4702169; doi:10.1038/srep18945)
Supplement: Supplementary Information [file srep18945-s1.doc]

***Supporting Information***

**Effects of** **calcitriol on random skin flap survival in rats**

Kai-liang Zhou^1^, Yi-hui Zhang^2^, Ding-sheng Lin^1^*, Xian-yao Tao^1^, Hua-zi Xu^1^

1, Department of Orthopaedic Surgery, The Second Affiliated Hospital of Wenzhou Medical University & The Second Clinical Medical College of Wenzhou Medical University, Wenzhou, China

2, Department of Traditional Chinese Medicine, The Second Affiliated Hospital of Wenzhou Medical University & The Second Clinical Medical College of Wenzhou Medical University, Wenzhou, China

Kai-Liang Zhou^1^, Yi-Hui Zhang^2^, Ding-Sheng Lin^1^*, Xian-Yao Tao^1^, Hua-zi Xu^1^ NO.109, XueYuan Road(West), LuCheng District, Wenzhou, ZheJiang Province, China ,325000

* Corresponding author: Ding-sheng Lin , Address:NO.109, XueYuan Road(West), LuCheng District , Wenzhou , ZheJiang Province, China telephone/facsimile numbers: 0086-577-88002760 Email:lindingsheng@gmail.com


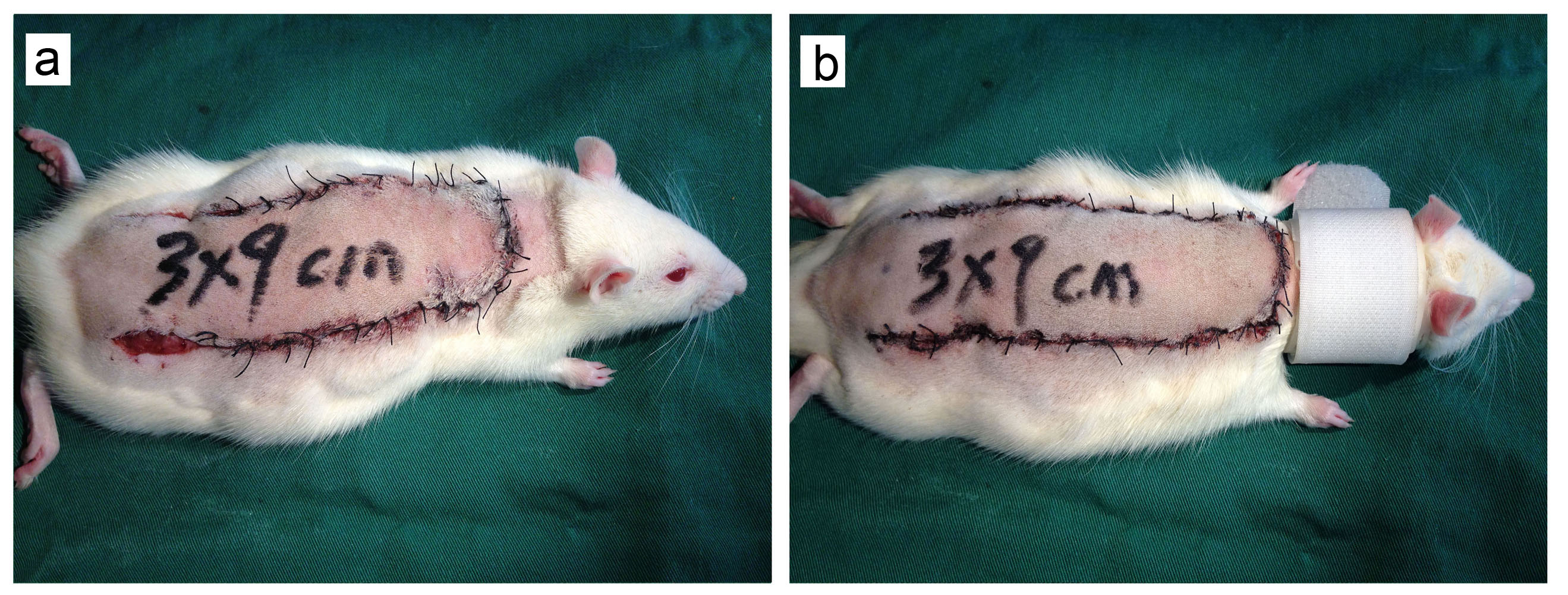
**Figure.S1 Rats were fitted with a neck collar to prevent self-mutilation.** (a) At the start of the experiment, authors found that rats had a self-mutilation tendency, and they would bite the random ﬂaps on their back and cause the ﬂaps injury. (b)The collar, which has applied to the chinese utility model patent (ZL 2014 2 0052396.X), is invented to prevent this phenomenon.

**We used a single membrane instead of the entire membrane to cover each target**

**protein respectively.**

**The full-length gel images for Fig.3e,** **Fig. 4e and** **Fig. 5d**

**Fig.3e**

**
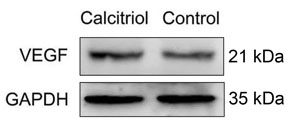
**

**Fig. 4e**

**
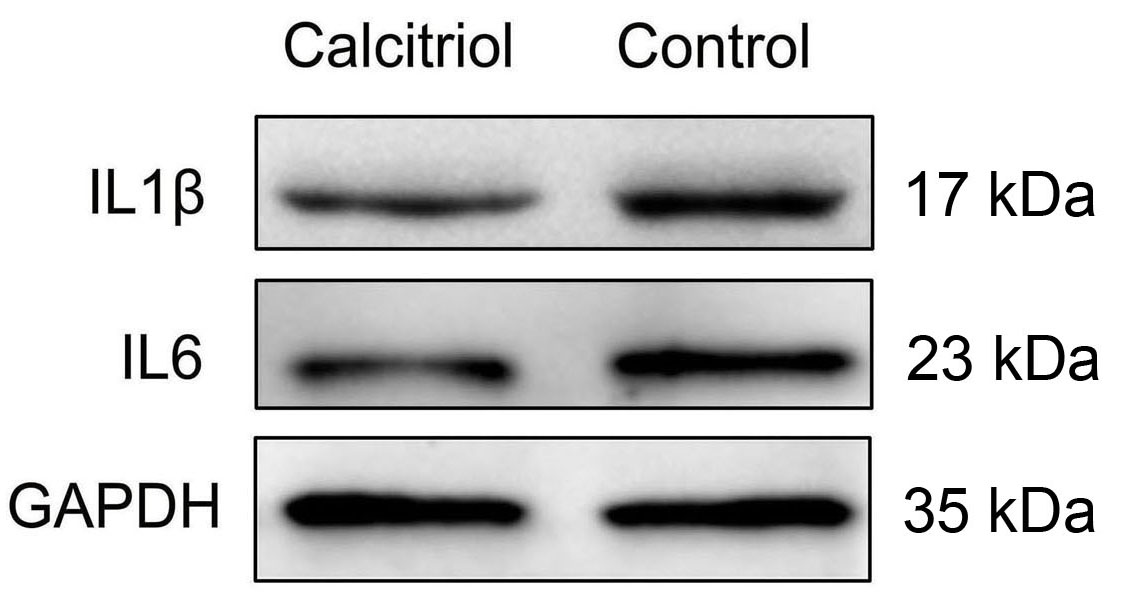
**

**Fig. 5d (The molecular weight of Beclin1 and p62 is quite close, so we cut the two from gels and incubated with respective primary antibodies.)**

**
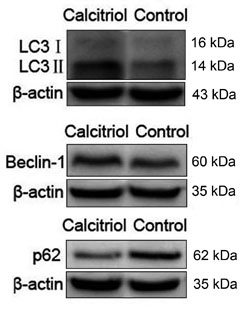
**
